# Large Language Model Morphology-to-Genotype Inference for Microsatellite Instability From Colorectal Carcinoma Histopathology Reports

**DOI:** 10.7759/cureus.110466

**Published:** 2026-06-08

**Authors:** Mimna V M

**Affiliations:** 1 Pathology, All India Institute of Medical Sciences, Gorakhpur, Gorakhpur, IND

**Keywords:** colorectal carcinoma, digital pathology, histopathology reports, large language models, medical artificial intelligence, microsatellite instability

## Abstract

Microsatellite instability (MSI) is an important prognostic and predictive biomarker in colorectal carcinoma and is associated with several characteristic histomorphological features. While large language models (LLMs) have been investigated for structured data extraction from histopathology reports, their ability to infer molecular genotype from text-based morphologic descriptions remains underexplored. This study evaluated structured data extraction, MSI prediction performance, confidence behavior, and biological reasoning quality of two LLMs using colorectal carcinoma pathology reports with The Cancer Genome Atlas (TCGA)-assigned MSI status as the reference standard. Fifty colorectal carcinoma reports, including 25 MSI-high (MSI-H) and 25 microsatellite-stable (MSS), from TCGA database were stratified by morphologic signal strength. ChatGPT 5.2 (OpenAI, San Francisco, CA) and Gemini 3 (Google, Mountain View, CA) were given an identical zero-shot prompt to extract 17 histopathologic variables across structural, staging, invasion, and MSI-associated morphologic categories, predict MSI status with confidence scores, and provide biological justification. Both models demonstrated high extraction accuracy for structural variables (85.0-88.5%) but substantially lower performance for MSI-associated morphologic variables (ChatGPT 5.2: 39.4%; Gemini 3: 43.9%), with mucinous differentiation showing the highest omission rate and signet ring cell morphology the lowest extraction accuracy. Overall, MSI prediction accuracy was 64.0% for Gemini 3 and 16.0% for ChatGPT 5.2. Among committed predictions, ChatGPT 5.2 demonstrated perfect specificity and positive predictive value, whereas Gemini 3 showed a morphologic signal-dependent accuracy gradient and a substantially higher rate of high-confidence incorrect predictions (20.0% versus 2.0%). General-purpose LLMs demonstrated variable ability to recognize MSI-associated histomorphologic descriptors, with systematic failures in extraction directly limiting downstream genotype inference.

## Introduction

Microsatellite instability (MSI) is recognized as a clinically significant molecular biomarker in colorectal carcinoma. MSI-high (MSI-H) tumors account for approximately 15-20% of sporadic colorectal cancers and represent a defining molecular feature of Lynch syndrome-associated carcinomas. It has prognostic, predictive, and hereditary cancer-screening significance, and its determination is incorporated into standard reporting protocols for resected colorectal carcinoma [1].

MSI-H colorectal carcinomas are characterized by a distinct constellation of histomorphological features, including right-sided anatomic location, mucinous or signet ring cell differentiation, medullary growth pattern, prominent tumor-infiltrating lymphocytes, and a peritumoral Crohn's-like lymphoid reaction [2-4]. These features underpin established clinicopathological scoring systems that triage cases for confirmatory molecular testing [5]. However, significant morphological overlap exists between MSI-H and microsatellite stable (MSS) tumors, particularly when classical features are limited or atypically present [6].

Computational approaches for predicting MSI status from histopathological data have advanced considerably over the past decade. Deep learning models trained on whole-slide images now achieve promising performance for MSI prediction directly from hematoxylin and eosin-stained sections, with several models implemented in clinical pre-screening workflows [7-9]. Concurrently, general-purpose large language models (LLMs) have demonstrated the ability to extract structured data from unstructured histopathology reports. For example, Truhn and colleagues demonstrated that GPT-4 could accurately extract staging and lymph node information from The Cancer Genome Atlas (TCGA) colorectal cancer reports in a zero-shot setting [10]. Subsequent studies have extended these structured extraction methods to breast, lung, and other cancer report types [11,12].

Despite rapid progress in these areas, a distinct question remains insufficiently explored. The ability of general-purpose LLMs to infer molecular genotype from text-based morphological descriptions, without access to the underlying histological substrate, has not been systematically characterized. This scenario is relevant to registry-based research, consultation practice, retrospective audits, and diagnostic settings with limited access to molecular testing. The performance of contemporary LLMs in this morphology-to-genotype inference task, when compared to genomic ground truth, has not been formally evaluated.

This study evaluated the performance of structured data extraction, MSI prediction accuracy, confidence behavior, and biological reasoning quality of two contemporary general-purpose LLMs, ChatGPT 5.2 (OpenAI, San Francisco, CA) and Gemini 3 (Google, Mountain View, CA), using 50 colorectal carcinoma histopathology reports from TCGA with molecularly confirmed MSI status as ground truth [13].

## Materials and methods

Study design

This was a retrospective observational evaluation study comparing the diagnostic performance of two general-purpose LLMs for colorectal carcinoma histopathology reports, using molecularly confirmed MSI status as the ground truth. The study used de-identified, publicly available pathology reports from TCGA [13].

Case selection

Fifty surgical pathology reports of colorectal adenocarcinoma were drawn from TCGA Colon Adenocarcinoma (TCGA-COAD) and TCGA Rectal Adenocarcinoma (TCGA-READ) cohorts, comprising 25 MSI-high and 25 MSS cases [13]. The molecular MSI status assigned by TCGA served as ground truth. Cases were selected to ensure the availability of complete pathology reports and to provide morphologic heterogeneity within each group. The full pathology report, including diagnosis, gross description, microscopic description, and any synoptic summary, was used as input for each case without any pre-processing.

Categorization of morphologic signal strength

For a stratified analysis of the LLM performance, each report was prospectively assigned to one of three morphologic signal strength groups according to the presence of MSI-discriminatory histological features, including mucinous or signet ring cell differentiation, medullary growth pattern, prominent tumor-infiltrating lymphocytes, and Crohn’s-like peritumoral lymphoid reaction. The categories were defined as follows: sparse, indicating no or minimal MSI-associated features; moderate, indicating one to two features; and strong, indicating multiple (>2) prominent MSI-associated features.

Assessment of large language models and prompt construction

Two contemporary general-purpose LLMs were evaluated: ChatGPT 5.2 and Gemini 3. Both models were accessed via their respective public web interfaces using default settings in January 2026. Each report was submitted to each model in a new, independent session to prevent contextual carry-over between cases.

An identical zero-shot prompt was applied to both models. The prompt directed each model to interpret the report and included two sequential tasks. The first task required the extraction of 17 structured variables across four categories: structural (histologic type, grade, location, size, configuration), staging (pT, pN, pM), invasion (lymphovascular invasion, surgical margins), and MSI-discriminatory (mucinous differentiation, tumor infiltrating lymphocytes, Crohn’s-like lymphoid reaction, signet ring cell component, tumor budding, additional morphologic features). LLMs were instructed to extract only stated information and to record “not reported” when a variable was absent. The second task required predicting MSI status (MSI-high, MSS, or indeterminate) based solely on morphological features. Each model was also asked to provide a confidence score on a 0-100 scale and a three- to five-sentence biological justification that references only features described in the report.

Outcome measures and error classification

**Figure 1:**
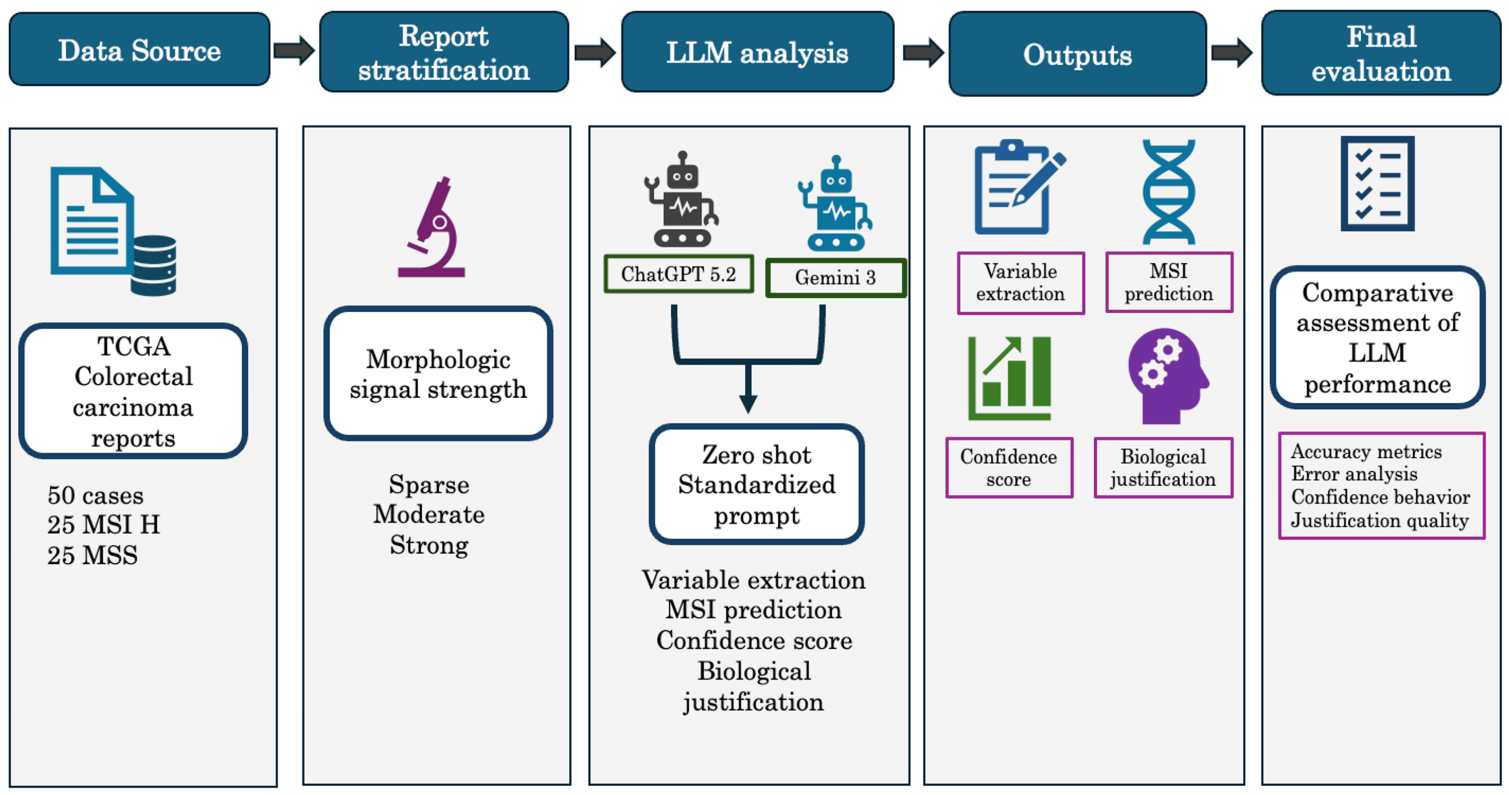
Study methodology and workflow. TCGA: The Cancer Genome Atlas; MSI: microsatellite instability; MSI-H: microsatellite instability-high; MSS: microsatellite stable; LLM: large language model. Figure created by the author using Microsoft PowerPoint (Microsoft Corporation, Redmond, WA).

For each extracted variable, model output was compared with the source pathology report and classified as correct (concordant with report text), omission (failure to extract a reported variable), or hallucination (assertion of a feature not present in the report). For MSI prediction, the model output was compared with TCGA-assigned ground truth. Diagnostic performance metrics, including accuracy, sensitivity, specificity, positive and negative predictive value, and F1 score, were calculated. Confidence behavior was assessed by comparing the mean confidence scores for correct and incorrect predictions and by quantifying the proportion of high-confidence incorrect predictions (confidence ≥70). Biological justifications were evaluated using a three-domain rubric: biological plausibility, fidelity to report content (use of only report-described features), and relevance to the predicted MSI status. Each domain was rated on a scale of 1 to 3, and the scores were summed to yield a composite score out of 9. The study methodology is represented in Figure 1.

Statistical analysis

Statistical analyses were performed using R software (version 4.5.2; R Foundation for Statistical Computing, Vienna, Austria).

## Results

Cohort characteristics

The study cohort comprised 50 colorectal adenocarcinoma cases, including 25 MSI-H and 25 MSS tumors. Among MSI-H tumors, 14 cases (56%) had a strong morphologic signal, eight (32%) had a moderate signal, and three (12%) had a sparse signal. In contrast, most MSS tumors demonstrated sparse morphologic signal (19, 76%), while moderate and strong morphologic signal patterns were observed in four (16%) and two (8%) cases, respectively. The two MSS tumors in the strong-signal category were morphologically discordant and exhibited prominent MSI-associated features.

Performance of structured data extraction

**Figure 2:**
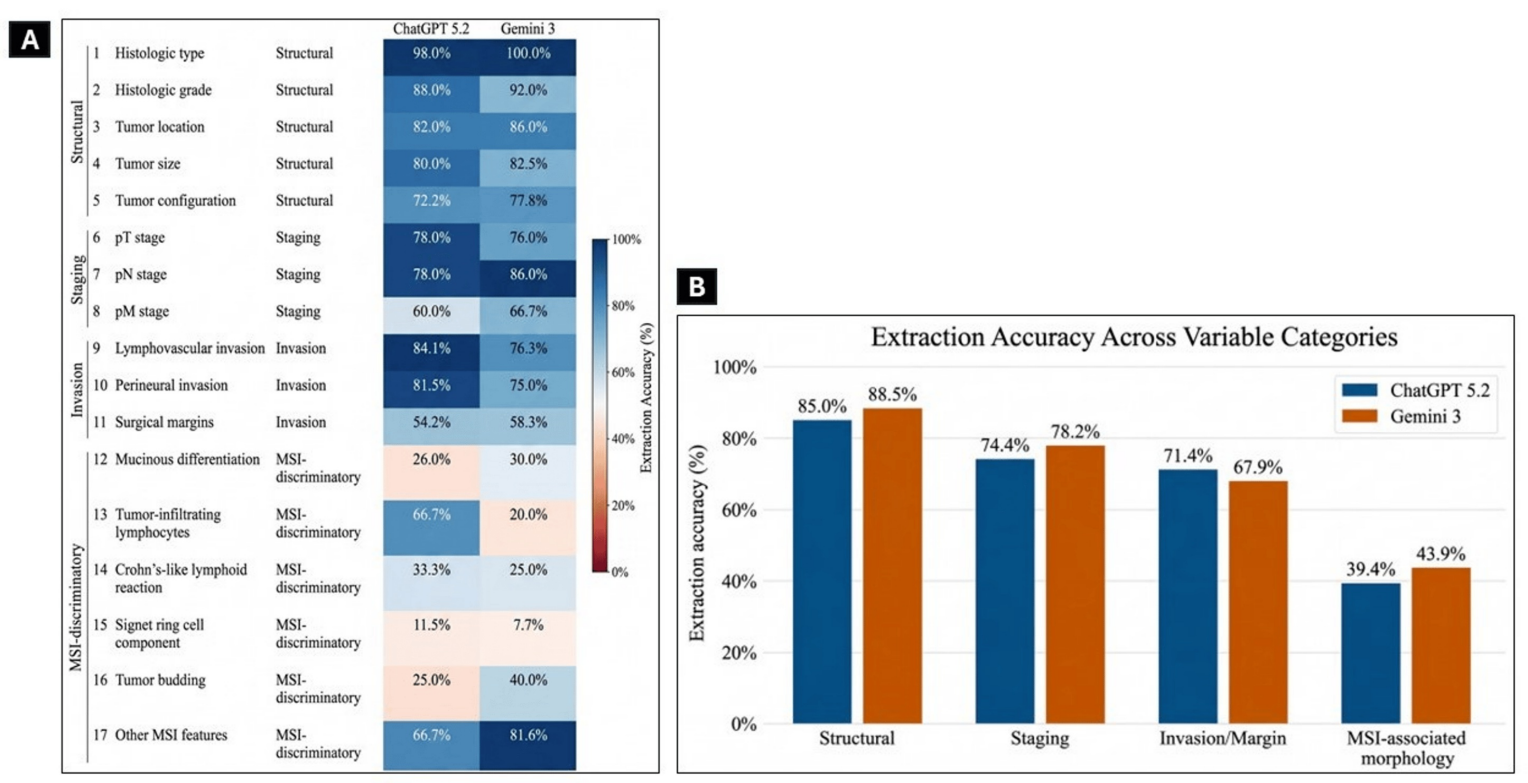
Extraction performance of ChatGPT 5.2 and Gemini 3 across histopathologic variable categories. (A) Heatmap demonstrating variable-level extraction accuracy across structural, staging, invasion-related, and MSI-discriminatory histopathologic variables. Both models demonstrated high extraction accuracy for structural and staging variables but substantially reduced performance for MSI-associated morphologic descriptors, particularly signet ring cell component and Crohn’s-like lymphoid reaction. (B) Grouped bar chart summarizing category-level extraction accuracy, demonstrating progressive reduction in extraction performance from structural variables to MSI-associated morphologic features in both models. MSI: microsatellite instability.

Both models achieved high extraction accuracy for routinely reported structural and staging variables; however, performance declined across more interpretive morphologic fields. Structural variables yielded the highest extraction accuracy, with ChatGPT 5.2 reaching 85.0% and Gemini 3 reaching 88.5%. Staging variables demonstrated accuracies of 74.4% and 78.2%, respectively. In contrast, extraction performance for MSI-associated morphologic variables was substantially lower, with accuracies of 39.4% for ChatGPT 5.2 and 43.9% for Gemini 3 (Figure 2).

Extraction failures most frequently involved morphologic descriptors conventionally associated with MSI. Mucinous differentiation accounted for the highest number of omissions, with omission rates of 36 out of 50 cases for ChatGPT 5.2 and 33 out of 50 cases for Gemini 3. Signet ring cell morphology exhibited the lowest extraction accuracy among all the evaluated variables, with accuracies of 11.5% and 7.7%, respectively. Crohn’s-like lymphoid reaction was inconsistently identified by both models. Tumor-infiltrating lymphocyte (TIL) extraction showed substantial inter-model variation, with ChatGPT 5.2 outperforming Gemini 3 (66.7% versus 20.0%) (Figure 2A).

Both models exhibited hallucination events, most notably in surgical margin assessment, where margin status was sometimes generated despite its absence in the source report. Neither model incorporated mismatch repair immunohistochemistry data during either extraction or prediction. Overall, extraction errors disproportionately affected MSI-associated morphologic variables, which directly limited the accuracy of downstream morphology-based MSI inference.

Overall MSI prediction performance

The two models exhibited distinct MSI prediction strategies and performance profiles. Across 50 cases, Gemini 3 achieved a prediction accuracy of 64.0% (32/50), while ChatGPT 5.2 attained an accuracy of 16.0% (8/50) (Table 1). This disparity was largely due to ChatGPT 5.2’s higher rate of indeterminate predictions in 76.0% of cases, compared to 8.0% for Gemini 3.

**Table 1:** Overall MSI prediction outcomes. MSI: microsatellite instability; MSI-H: microsatellite instability-high; MSS: microsatellite stable; LLM: large language model.

LLM response	ChatGPT 5.2		Gemini 3	
	MSI-H (n = 25)	MSS (n = 25)	MSI-H (n = 25)	MSS (n = 25)
Correct prediction	5 (20.0%)	3 (12.0%)	17 (68.0%)	15 (60.0%)
Incorrect prediction	4 (16.0%)	0 (0.0%)	7 (28.0%)	7 (28.0%)
Indeterminate	16 (64.0%)	22 (88.0%)	1 (4.0%)	3 (12.0%)
Overall accuracy	16.0%		64.0%	

In the MSI-H cohort, Gemini 3 correctly identified 17 of 25 cases (68.0%), while ChatGPT 5.2 correctly classified five of 25 cases (20.0%). ChatGPT 5.2 produced no false-positive predictions (zero MSS cases incorrectly classified as MSI-H), although it frequently abstained from classification. In contrast, Gemini 3 provided broader classification coverage, though at a higher false-positive rate. When considering only decided cases, ChatGPT 5.2 achieved perfect specificity and positive predictive value, whereas Gemini 3 demonstrated balanced sensitivity and specificity with a substantially lower abstention rate (Table 2).

**Table 2:** Diagnostic performance metrics on decided cases.

Metric	ChatGPT 5.2 (n = 12, decided cases)	Gemini 3 (n = 46, decided cases)
Sensitivity	55.6%	70.8%
Specificity	100%	68.2%
Positive predictive value	100%	70.8%
Negative predictive value	42.9%	68.2%
F1-score	0.714	0.708
Accuracy on decided cases	66.7%	69.6%

These results indicate that ChatGPT 5.2 employs a conservative, precision-focused prediction strategy, whereas Gemini 3 adopts a broader, coverage-oriented approach.

Effect of morphologic signal strength on MSI prediction

MSI prediction performance exhibited a clear association with morphologic signal strength, especially for Gemini 3. Prediction accuracy for Gemini 3 increased incrementally from sparse to strong morphologic signal categories, reaching its highest accuracy in strong-signal cases, with no indeterminate responses (Table 3). In contrast, ChatGPT 5.2 maintained a predominantly conservative approach across all signal categories, with consistently high indeterminate rates despite only modest improvement in prediction accuracy within the strong-signal group.

**Table 3:** MSI prediction performance stratified by morphologic signal strength. * Strong category includes morphologically discordant MSS tumors with prominent MSI-associated features. MSI: microsatellite instability; MSS: microsatellite stable.

Morphologic signal category	n	ChatGPT 5.2 prediction, n (%)			Gemini 3 prediction, n (%)		
		Correct	Incorrect	Indeterminate	Correct	Incorrect	Indeterminate
Sparse	22	3 (13.6)	2 (9.1)	17 (77.3)	15 (68.2)	4 (18.2)	3 (13.6)
Moderate	12	1 (8.3)	0 (0.0)	11 (91.7)	6 (50.0)	5 (41.7)	1 (8.3)
Strong*	16	4 (25.0)	2 (12.5)	10 (62.5)	11 (68.8)	5 (31.2)	0 (0.0)

Two MSS tumors exhibiting prominent mucinous morphology constituted morphologically discordant cases within the strong-signal category. Gemini 3 classified both as MSI-H, while ChatGPT 5.2 produced indeterminate predictions for both cases. These findings indicate that isolated MSI-associated morphologic features, particularly mucinous differentiation, may disproportionately affect morphology-based genotype inference when additional contextual morphologic features are lacking.

Confidence behavior and biological justification quality

**Figure 3:**
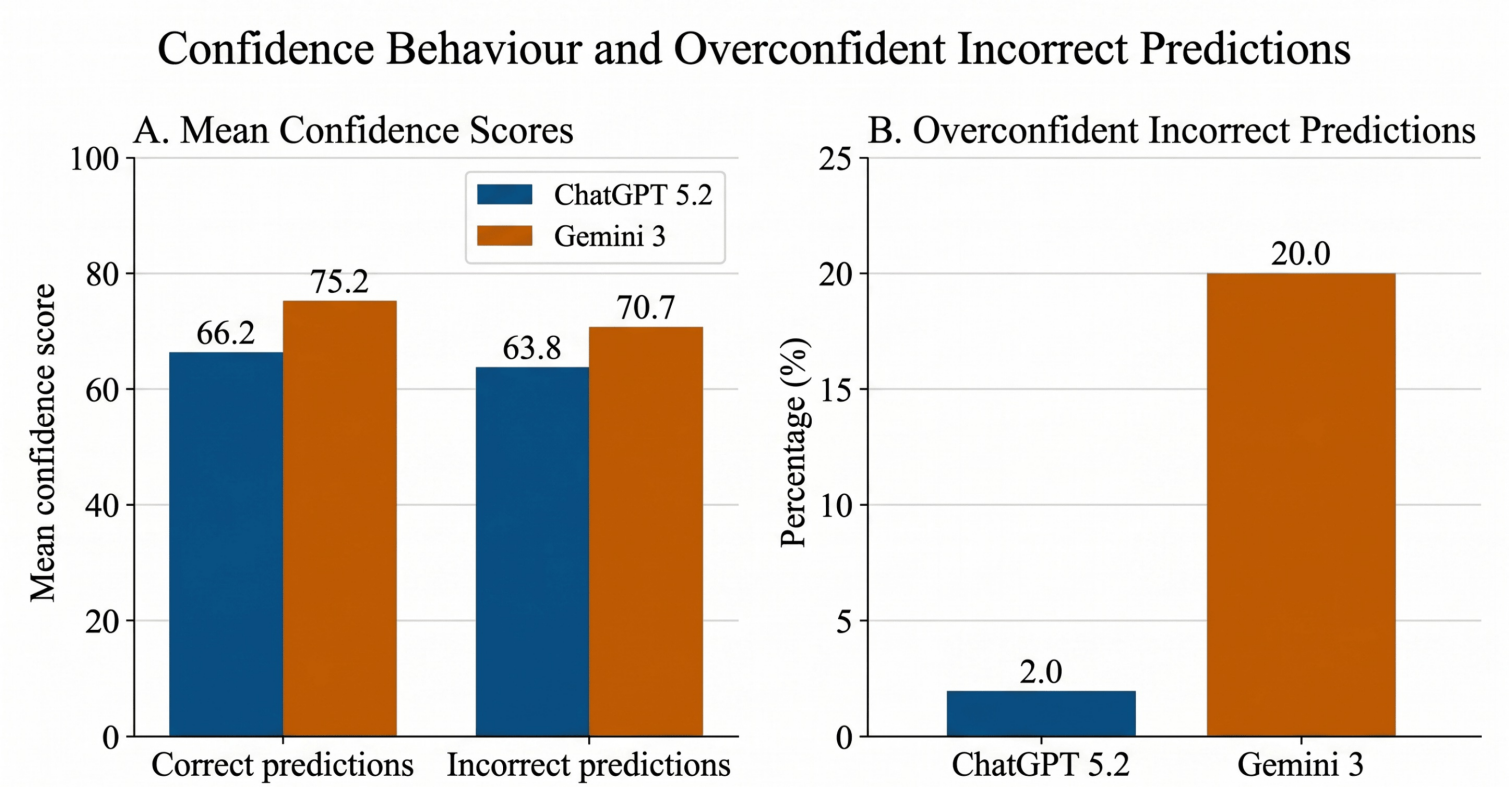
Confidence behavior of ChatGPT 5.2 and Gemini 3 during MSI prediction. (A) Mean confidence scores for correct and incorrect predictions in both models, demonstrating limited separation between accurate and erroneous predictions at the confidence level. (B) Proportion of overconfident incorrect predictions, showing a substantially higher rate in Gemini 3 compared with ChatGPT 5.2. MSI: microsatellite instability.

The two models exhibited distinct confidence behaviors during MSI prediction. ChatGPT 5.2 assigned substantially lower overall confidence scores, reflecting a predominant use of indeterminate responses. In contrast, Gemini 3 consistently generated higher-confidence definitive predictions. The mean confidence scores for correct and incorrect predictions demonstrated only limited separation in both models, indicating relatively poor discrimination between accurate and erroneous predictions at the confidence level (Figure 3A).

Gemini 3 exhibited a substantially higher rate of high-confidence incorrect predictions compared with ChatGPT 5.2. Among incorrect predictions, 71.4% of Gemini 3 errors received confidence scores of 70 or higher, resulting in an overall overconfident error rate of 20.0%. In contrast, ChatGPT 5.2 produced only a single high-confidence incorrect prediction (2.0%) (Figure 3B).

Both models generated biologically plausible justifications in the majority of cases. ChatGPT 5.2 achieved a higher mean composite justification score than Gemini 3 (8.7/9 versus 8.1/9) and produced a greater proportion of high-quality justifications (86% versus 74%). Moderate-quality justifications were more frequent in Gemini 3 (26%) compared with ChatGPT 5.2 (12%), while poor-quality justifications were uncommon in both models. The primary limitation of ChatGPT 5.2 was insufficient information in morphologically sparse reports, whereas Gemini 3 more frequently demonstrated interpretive overreach by incorporating probabilistic reasoning or clinicopathologic assumptions not explicitly supported by the report text.

## Discussion

This study examined two related applications of general-purpose LLMs for assessing colorectal carcinoma pathology reports: structured extraction of morphologic information and inference of MSI status from morphology. Joint evaluation of these tasks revealed that the accuracy of downstream MSI prediction was directly limited by the extraction of MSI-associated morphologic features. Although both ChatGPT 5.2 and Gemini 3 were evaluated with the same prompt, the models employed substantially different prediction strategies, resulting in distinct performance outcomes and confidence profiles, consistent with previous studies [14].

Both models achieved high extraction accuracy for routinely reported structural and staging variables, but exhibited substantially lower performance for MSI-associated morphologic features. Variables such as histologic type, tumor location, and pathologic stage are generally represented by standardized categorical terms, facilitating their extraction from pathology reports. In contrast, MSI-associated morphologic descriptors, including mucinous differentiation, signet ring morphology, Crohn’s-like lymphoid reaction, and tumor-infiltrating lymphocytes, are often described using interpretive or qualified language. Recognition of these features frequently relies on contextual wording and subjective descriptive thresholds rather than fixed terminology. This reliance likely contributed to the observed decline in extraction accuracy for these variables. Previous studies evaluating LLM-based analysis of pathology reports have reported similar challenges in extracting nuanced morphologic or descriptive information from unstructured pathology text [11,15,16]. These findings indicate that variability in the language of morphologic reporting remains a significant limitation to downstream morphology-based molecular inference. Increased standardization in reporting MSI-associated morphologic features, together with prompt strategies that emphasize detailed morphologic interpretation, may enhance future LLM-assisted pathology text analysis [17,18].

Hallucination events were identified in both models, most commonly during surgical margin assessment, in which margin status was occasionally generated despite the absence of documentation in the source report. This pattern appeared to reflect inference based on anticipated report structure rather than direct extraction from report text [19,20]. Importantly, unsupported molecular or immunohistochemical inference was not identified in either model.

The two models demonstrated markedly different MSI prediction behaviors. ChatGPT 5.2 adopted a conservative approach characterized by frequent indeterminate responses, whereas Gemini 3 generated substantially broader decisional coverage with higher overall accuracy. However, Gemini 3 also demonstrated a higher frequency of high-confidence incorrect predictions, particularly in morphologically discordant cases. Both MSS tumors with prominent mucinous morphology were classified as MSI-H by Gemini 3, whereas ChatGPT 5.2 returned indeterminate predictions. Although mucinous differentiation is strongly associated with MSI-H colorectal carcinoma, it is not specific and may also occur in MSS tumors [21]. These findings suggest that broader coverage-oriented prediction strategies may overemphasize isolated MSI-associated morphologic features when additional contextual findings are limited.

Prediction performance in Gemini 3 additionally demonstrated a relationship with morphologic signal strength, with increasing accuracy from sparse- to strong-signal categories. This trend parallels established morphology-based approaches used in routine pathology practice, where confidence in MSI prediction increases when multiple characteristic morphologic features are present [5]. However, the systematic under-extraction of MSI-associated morphologic variables in both models directly limited the ceiling of downstream morphology-based MSI inference. Future studies incorporating structural and staging variables, such as tumor location and pathologic stage, alongside morphologic descriptors, may improve MSI prediction performance and better reflect the integrative reasoning applied in routine pathology practice.

Several limitations must be acknowledged when interpreting these findings. The study cohort was relatively small and sourced exclusively from TCGA pathology reports, which may differ in structure and descriptive style from routine diagnostic reports. Only two general-purpose LLMs were evaluated at a single time point, and model behavior may change with future versions and updates. Furthermore, a single zero-shot prompt strategy was employed, and prompt design may affect both extraction and prediction performance [17]. Morphologic signal categorization and extraction accuracy assessment were conducted by a single observer without independent verification or inter-rater reliability measurement. The absence of a second observer is a methodological limitation, as both tasks involve interpretive judgment, particularly in assigning morphologic signal-strength categories and classifying extraction outputs as correct, omitted, or hallucinated. In addition, hallucination events were identified but not systematically quantified; formal enumeration and subtype classification of hallucinations would strengthen future evaluations of LLM reliability in pathology report analysis.

## Conclusions

General-purpose LLMs have demonstrated the capability to infer MSI from free-text colorectal carcinoma pathology reports. However, their performance is limited by incomplete recognition of morphologic features associated with MSI. The findings indicate that effective morphology-to-genotype inference requires both advanced model reasoning and precise extraction of biologically relevant histopathologic information. These results underscore the potential and current limitations of LLMs in extracting and interpreting morphological signals associated with underlying molecular alterations.
